# Big Data-Driven Evolution of a Diagnostic Multiplex IgE-Test: Enhancing Accuracy and Efficacy in Allergy Diagnostics

**DOI:** 10.3390/ijms26094249

**Published:** 2025-04-29

**Authors:** Christian Lupinek, Peter Forstenlechner, Anna Ringauf, Raffaela Campana, Artan Salihu, Martina Aumayr, Irene Mittermann

**Affiliations:** MacroArray Diagnostics GmbH, Lemboeckgasse 59/4, 1230 Vienna, Austria

**Keywords:** microarray, macroarray, multiplex, allergy, test, diagnosis, IgE-sensitisation, molecular allergology, big-data, ALEX

## Abstract

The ALEX^2^-test (MacroArray Diagnostics, Vienna, Austria) is a diagnostic multiplex IgE-test for the simultaneous detection of IgE to 178 allergens and 117 extracts, in addition to total IgE. Test results from more than 90 countries are stored on a GDPR-compliant cloud server for backup, customer support, and continuous postmarket surveillance. To improve the coverage of exposomes on a global scale and to further increase the sensitivity of the test, the allergen panel was updated from ALEX^2^ to ALEX^3^. By mid-2023, when ALEX^3^ was designed, almost 400,000 real-world ALEX^2^ test results were available. Analysing prevalences and average sIgE-levels of individual allergen preparations, coverage of extracts by components, and co-reactivity of members of the same allergen family provided a rationale for updating the array. In parallel, based on the scientific literature and clinical studies, new allergens were selected. On ALEX^3^, 218 allergens and 82 extracts will be represented, including 52 new allergens. Allergen preparations with low prevalence and clinical relevance, as well as redundant allergens and extracts, were discontinued. New allergens encompass, e.g., cyclophilins, alpha-gal, and additional markers from respiratory and food allergen sources. Using a large dataset of ALEX^2^ test results exemplifies the targeted, data-driven improvement of a diagnostic IgE-macroarray.

## 1. Introduction

Detection of allergen-specific IgE (sIgE) is a cornerstone of allergy diagnosis by confirming or excluding allergic sensitisation to a broad range of allergen sources [1]. However, the use of allergen extracts, both for the detection of allergen-specific IgE and for skin prick testing, conveys considerable diagnostic uncertainty, due to significant variability in the composition and allergen concentration of the respective extracts. This inconsistency is not limited to products from different manufacturers but was also observed for different batches from the same manufacturer [2,3,4,5,6,7]. Even in the case of strict adherence to the same extraction protocol, the heterogeneous quality of the raw material can account for a substantial increase or drop in allergen concentration [8]. Sustained attempts to standardise extracts did not succeed in overcoming this constraint [9]. A conceptual limitation of extract-based diagnostics is the inability to distinguish multiple genuine sensitisations from cross-sensitisations. By the use of allergen molecules (also referred to as “components”) instead of extracts, both limitations could be overcome [10], leading to improved accuracy of allergy diagnosis and immunotherapy prescription [11,12].

The combination of the molecular allergology approach with protein micro- or macroarray technology culminated in the development of IgE macroarrays for the multiplexed detection of allergen-specific IgE [13,14]. IgE macroarrays provide comprehensive information on the patients’ IgE-reactivity patterns in one step, usually to more than 100 allergens, consuming only minute volumes of samples. Such tests facilitate the differentiation between genuine and cross-sensitisations, they capture IgE-reactivities to uncommon allergen sources and, thus, can help to speed up the diagnostic workup [15]. However, for high diagnostic sensitivity of the test, it is mandatory that the allergens on the array cover those allergens which are prevalent in the respective area.

The ALEX^2^-test (Allergy Xplorer, MacroArray Diagnostics, Vienna, Austria) is an IgE macroarray featuring the currently most comprehensive panel of allergen molecules (n = 178) and extracts (n = 117) on the same test—in total 295 specificities, in addition to total IgE. It is currently used for allergy diagnosis in more than 90 countries worldwide. Recently, a new version of the test, ALEX^3^, was developed. The aims of this update of the ALEX-test were (1) to further improve the coverage of exposomes on a global scale, and (2) to replace selected extracts with defined natural or recombinant allergen molecules. For an IgE macroarray that is used globally, it is particularly challenging to ensure that a broad variety of exposomes [16] is covered as completely as possible. The latter frequently exhibit substantial differences, e.g., between distinct climate zones [17], but even between different countries of similar climatic conditions [18]. Therefore, only nine years after version one, the third version of ALEX is about to be launched, translating the growing knowledge about regional exposomes into the test’s layout. In the present paper, we describe and exemplify how a large set of real-world ALEX^2^ test results was analysed and provided a rationale to decide which allergen molecules and extracts are to be contained on the updated version of the ALEX-test and which ones convey redundant information and, thus, could be removed. In addition, examples of new allergens on ALEX^3^ are provided, together with a brief discussion of their relevance and role in the updated test. The aim of this article is to illustrate the data- and science-driven approach for the further improvement of a diagnostic sIgE-test.

**Remark 1:**  *There is no clear distinction between micro- and macroarrays. Commonly, multiplex tests with larger spots which are easily discernible to the naked eye are referred to as macroarrays.*

**Remark 2:**  *Throughout this article, the standardised WHO/IUIS nomenclature of allergens will be used, as reviewed in [19].*

## 2. Results

### 2.1. Extracts and Allergen Molecules of the Same Source Contained on ALEX^2^

#### 2.1.1. Example of Retaining the Extract: Spreading Pellitory (*Parietaria judaica*)

Spreading pellitory, also commonly known as pellitory-of-the-wall or wall pellitory, is a weed occurring throughout Europe, but with particularly high prevalence in the Mediterranean region. In this area, it was described as one of the most important sources eliciting IgE-sensitisation and allergy symptoms [20]. In *Parietaria*, two non-specific lipid transfer proteins (nsLTP), i.e., Par j 1 and 2, had been identified as species-specific major allergens with highly overlapping IgE epitopes [21]. Therefore, in addition to the extract, Par j 2 is contained on ALEX^2^ as a specific marker allergen for allergic sensitisation to pollen from spreading pellitory.

**Figure 1:**
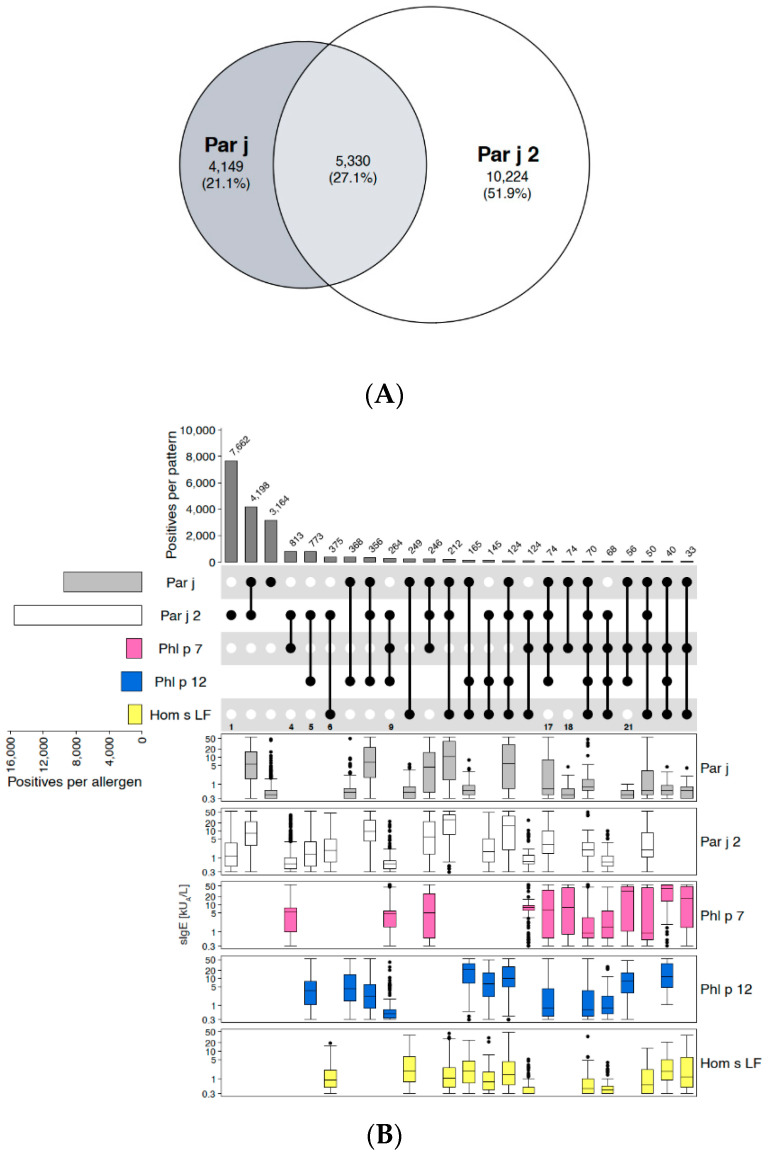
Spreading pellitory (*Parietaria judaica*). (**A**) Euler-plot of all samples positive to the extract (“Par j”) or to one of the main marker allergens from *Parietaria judaica*, Par j 2. Absolute numbers and percentages of cases are indicated for each respective segment. (**B**) Upset-plot (top), showing different reactivity patterns to Par j extract, Par j 2, and surrogate markers of the pan-allergen families of polcalcins (Phl p 7) and profilins (Phl p 12), as well as to a CCD-marker (Hom s LF). Numbers of cases for each individual allergen are indicated by bars on the left (“Positives per allergen”) and for each combination of IgE-reactivity by bars on top of the plot (“Positives per pattern”). The combination matrix illustrates patterns of reactivity: a single spot represents samples monoreactive to the respective allergen on the left, two spots that are connected by a vertical line show samples with IgE-reactivity to the two corresponding allergens on the left side, etc. To facilitate readability, every second line is shown in light grey; white dots display the absence of reactivity. At the bottom of the combination matrix, select columns mentioned in the text are indicated by numbers. Box-plots underneath each individual reactivity pattern show IgE-levels (kU_A_/L) to each of the abovementioned allergen-preparations (y-axes in log_10_ scale).

For our analysis, 19,703 real-world ALEX^2^ test results with IgE-reactivity to *Parietaria*-extract and/or to Par j 2 were used. The Euler-plot (Figure 1A) revealed only 27% of co-reactivity between extract and component, while the majority of samples (52%) were positive to Par j 2 but negative to the extract. Of all samples that had tested positive for the extract (n = 9479), 44% were negative to Par j 2, obviously due to IgE to other allergens than Par j 2 which are represented in the extract. The upset-plot in Figure 1B showed that among these sera, 3164 (33.4% of all extract-positive samples) were negative to pan-allergens (i.e., profilins and polcalcins, represented by the respective surrogate markers [see section on profilins] Phl p 12 and Phl p 7 from timothy grass) and cross-reactive carbohydrate determinants (CCD [22], on the ALEX-test, CCD-bearing human lactoferrin [Hom s LF] is used as CCD-marker) as well. Although average sIgE-levels to Par j-extract in this subgroup were relatively low (median: 0.4 kU_A_/L, Q1: 0.3 kU_A_/L, Q3: 0.6 kU_A_/L), the high number of samples exclusively positive to the extract which could not be explained by IgE-reactivity to pan-allergens on ALEX^2^ led to the decision to retain the extract.

Those cases that were positive to Par j 2 and negative to Par j-extract are due to the lower content of Par j 2 in the extract compared to the preparation of the pure component. Therefore, at low levels of IgE specific to Par j 2, the extract might remain negative, as is shown in Figure 1B (e.g., columns 1, 4–6 and 9). The same was observed for IgE-levels to pan-allergens which need to exceed a certain threshold to become detectable in the extract. In column nine, for example, sIgE to Par j 2, Phl p 7 and Phl p 12 taken together were too low to give rise to a positive signal for the extract, while in columns 17, 18 or 21, sIgE levels to the components were higher, giving rise to a positive signal to the extract, in column 18 even only driven by polcalcin.

#### 2.1.2. Example of Discontinuation of the Extract: Ribwort Plantain (*Plantago lanceolata*)

In analogy to spreading pellitory, ALEX^2^ contains both the extract and a marker allergen (Pla l 1) from ribwort plantain. Pla l 1 belongs to the family of Ole e 1-related proteins. Due to its limited cross-reactivity with other members of that family (also refer to the section on Ole e 1-like proteins below), it is used as a specific marker for sensitisation to ribwort plantain.

**Figure 2:**
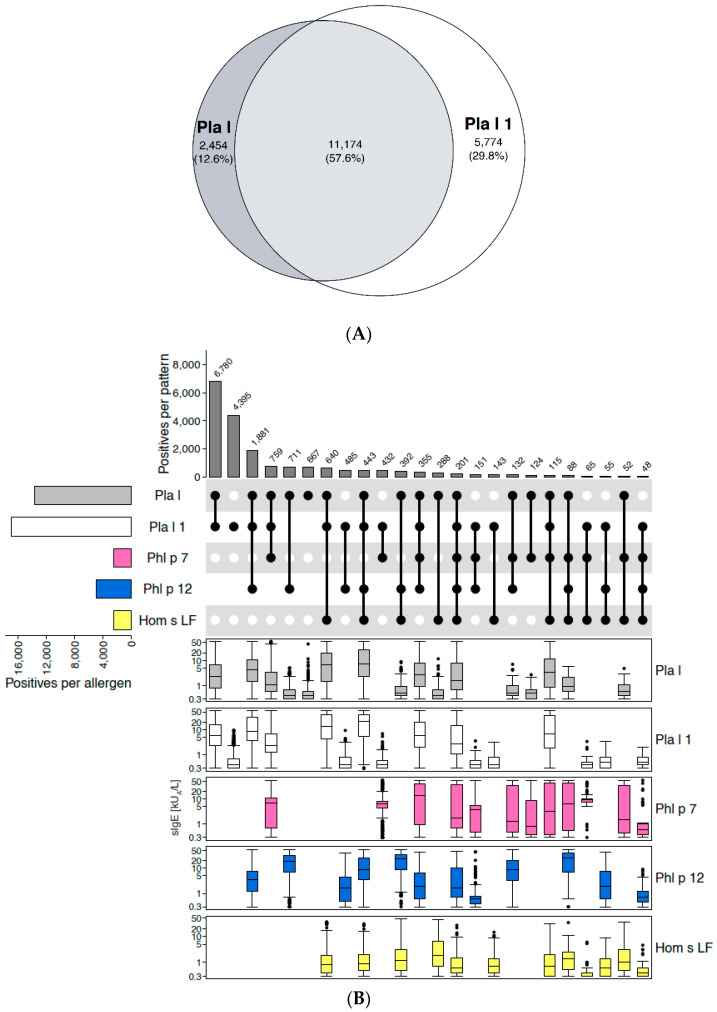
Ribwort plantain (*Plantago lanceolata*). (**A**) Euler-plot of all samples positive to the extract (“Pla l”) or to the main marker allergen from ribwort, Pla l 1. Absolute numbers and percentages of cases are indicated for each respective segment. (**B**) Upset-plot (top), showing different reactivity patterns to Pla l extract, Pla l 1, and surrogate markers of the pan-allergen families of polcalcins (Phl p 7) and profilins (Phl p 12), as well as to a CCD-marker (Hom s LF). Numbers of cases for each individual allergen are indicated by bars on the left (“Positives per allergen”) and for each combination of IgE-reactivity by bars on top of the plot (“Positives per pattern”). The combination matrix illustrates patterns of reactivity: a single spot represents samples monoreactive to the respective allergen on the left, two spots that are connected by a vertical line show samples with IgE-reactivity to the two corresponding allergens on the left side, etc. To facilitate readability, every second line is shown in light grey; white dots display absence of reactivity. Box-plots underneath each individual reactivity pattern show IgE-levels (kU_A_/L) to each of the abovementioned allergen-preparations (y-axes in log_10_ scale).

The Euler-plot (Figure 2A), which was based on a total of 19,402 ALEX^2^ test results positive to Pla l-extract and/or Pla l 1, revealed much more pronounced co-reactivity (58%) to both the extract and the component, as compared to the abovementioned example of spreading pellitory. Of all samples positive to Pla l-extract (n = 13,628), only 18% (n = 2454) were negative to Pla l 1, and 4.9% (667) were negative to Pla l 1, profilins, polcalcins and CCDs (Figure 2B). Due to this low rate of single-positives and because sIgE-levels to Pla l-extract in this group were low (median: 0.4 kU_A_/L, Q1: 0.3 kU_A_/L, Q3: 0.6 kU_A_/L), Pla l-extract was decided not to be included on ALEX^3^.

### 2.2. Allergen Families

#### 2.2.1. Example of Retaining and Adding Allergen Molecules: Storage Proteins (2S Albumins, 7S Globulins [Vicilins], 11S Globulins [Legumins])

**Figure 3:**
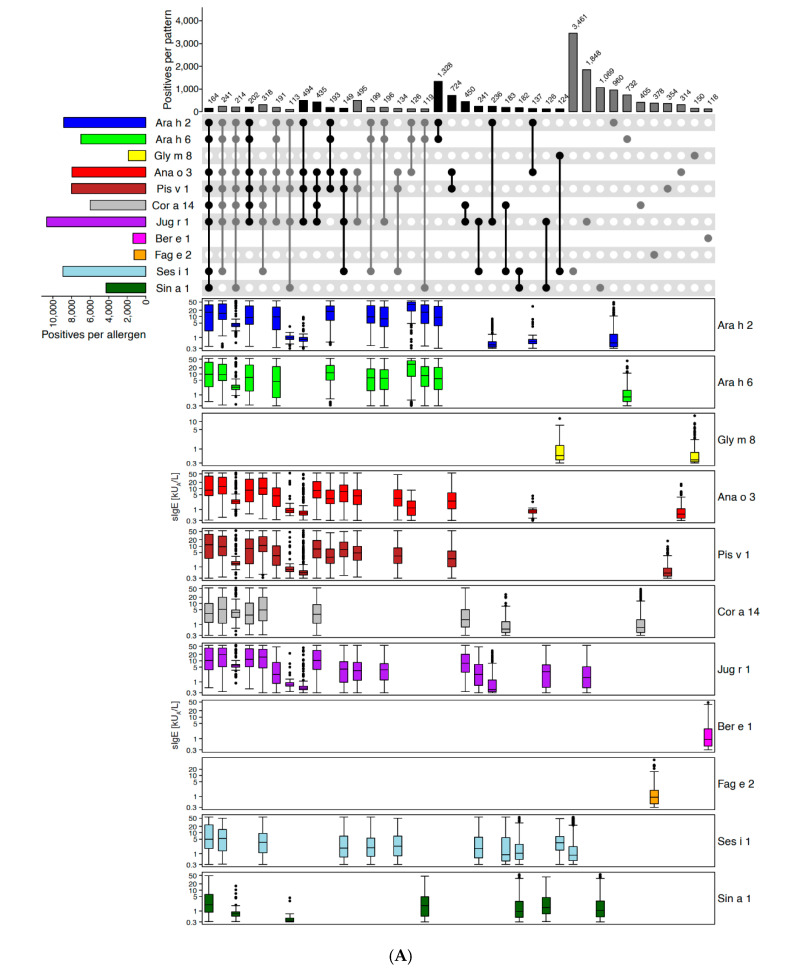

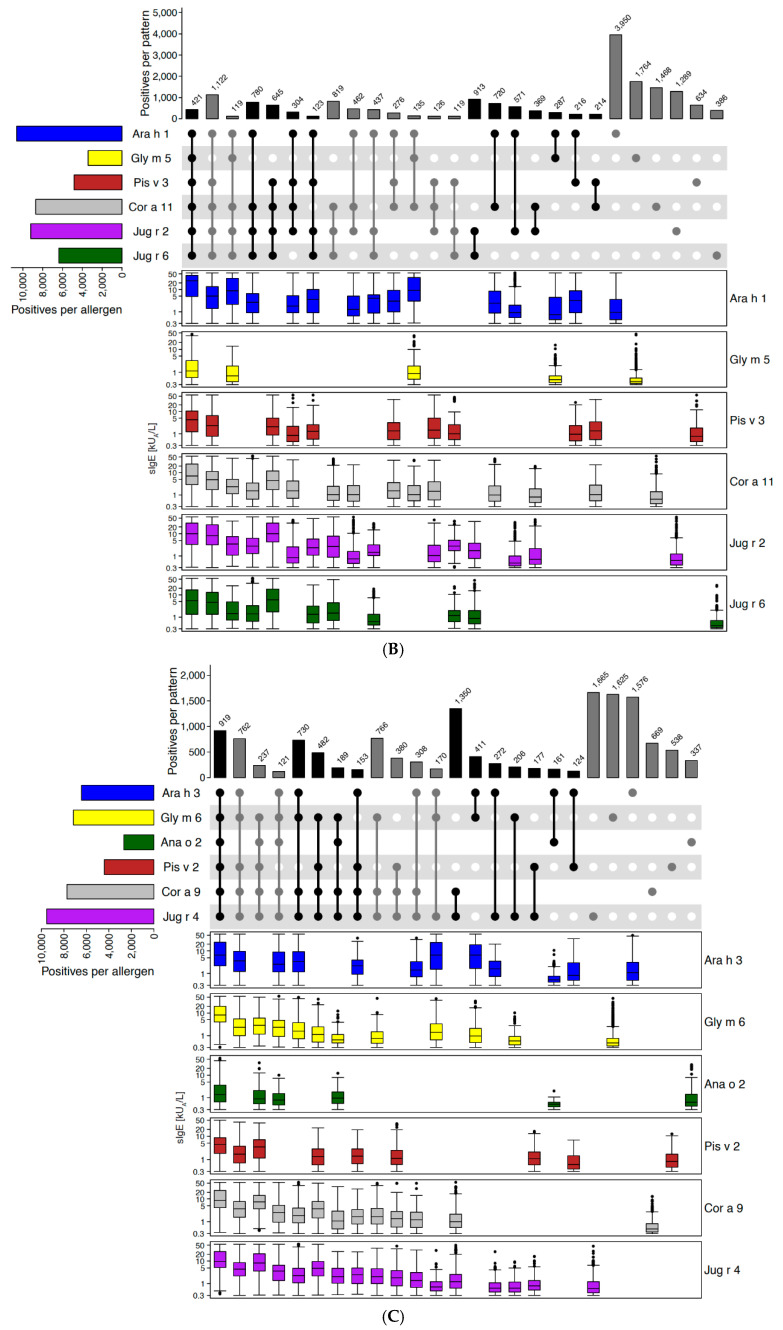
Storage protein families. Upset-plots (top), showing frequencies of reactivity patterns, and box-plots underneath each individual pattern showing IgE-levels (kU_A_/L, y-axes in log_10_ scale) to (**A**) 2S albumins, (**B**) 7S globulins and (**C**) 11S globulins on the ALEX^2^-test. Numbers of cases for each individual allergen are indicated by bars on the left (“Positives per allergen”) and for each combination of IgE-reactivity by bars on top of the Upset-plot (“Positives per pattern”). The combination matrix illustrates patterns of reactivity: a single dark (i.e., black or dark grey) dot represents samples monoreactive to the respective allergen on the left, two dark dots that are connected by a vertical line show samples with IgE-reactivity to the two corresponding allergens on the left side, etc. To facilitate readability, every second line is shown in light grey; white dots display absence of reactivity. Groups of reactivities to the same number of allergens are sorted from left (highest number of co-reactivities) to right (monoreactivities), each group either displayed by black or grey bars and dots, to facilitate identification of groups.

For the three different families of storage proteins, a total of 24,500 ALEX^2^ cases positive to at least one of the eleven 2S albumins on the ALEX^2^-test were analysed, 19,800 corresponding cases for the six vicilins, and 15,650 cases for the six legumins. Both reactivity patterns and allergen-specific IgE-levels were shown for all three families (Figure 3A–C). Results revealed that monosensitisations were the most common sensitisation patterns with, however, substantially lower sIgE-levels for most allergens when compared to samples exhibiting co-sensitisation to two or more allergens of the respective family (Figure 3A–C, upset-plots on top, right part of the charts). Of note, no co-reactivity with any other 2S albumin was observed for Fag e 2 from buckwheat and for Ber e 1 from Brazil nut (Figure 3A). This observation suggests that for most storage proteins, both shared and specific epitopes exist that might account for different combinations of co-reactivity and monoreactivities, respectively.

Average sIgE-levels were consistently higher for sera with broader reactivity profiles (Figure 3, columns on the left of each chart) which, however, were observed much less frequently than monosensitisations for 2S albumins and vicilins (Figure 3A,B).

Due to the highly diverse combinations of IgE-reactivity to storage proteins in general, and the predominance of monosensitisations across all three families (despite low average levels of IgE-reactivity in the latter group), no surrogate markers could be identified for the three storage protein families. Hence, all storage proteins represented on ALEX^2^ were kept on ALEX^3^, and additional members of those families were added (Table S1).

#### 2.2.2. Example of Removing and Adding Allergen Molecules: PR-10 Proteins

**Figure 4:**
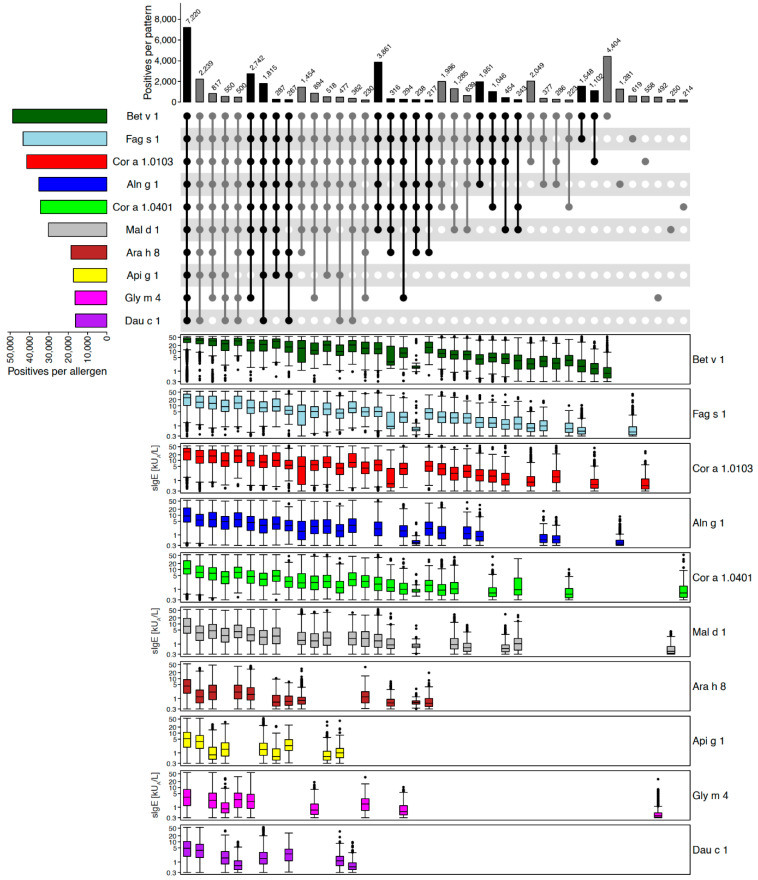
PR-10 allergens. Upset-plot (top), showing frequencies of reactivity patterns, and box-plots underneath each individual pattern showing IgE-levels (kU_A_/L, y-axes in log_10_ scale) to PR-10 allergens on the ALEX^2^-test. Numbers of cases for each individual allergen are indicated by bars on the left (“Positives per allergen”), and for each combination of IgE-reactivity by bars on top of the Upset-plot (“Positives per pattern”). The combination matrix illustrates patterns of reactivity: a single dark (i.e., black or dark grey) dot represents samples monoreactive to the respective allergen on the left, two dark dots that are connected by a vertical line show samples with IgE-reactivity to the two corresponding allergens on the left side, etc. To facilitate readability, every second line is shown in light grey; white dots display absence of reactivity. Groups of reactivities to the same number of allergens are sorted from left (highest number of co-reactivities) to right (monoreactivities), each group either displayed by black or grey bars and dots, to facilitate identification of groups.

In contrast to the previous example of storage proteins, PR-10 allergens exhibited pronounced mutual co-reactivity (Figure 4). As an example, among a total of 54,500 ALEX^2^ test results positive for at least one of the ten PR-10 allergens on the array, 48,850 (90%) were positive for Bet v 1 from silver birch. Average sIgE-levels declined from test results with reactivity to all ten allergens, towards monosensitised samples (Figure 4, left to right). Due to this apparent redundancy of coverage of PR-10 allergens by the ALEX^2^ array, Fag s 1 from beech (median: 0.49 kU_A_/L, Q1: 0.36, Q3: 0.9 kU_A_/L) and Cor a 1.0103 from hazel pollen (median: 0.54 kU_A_/L, Q1: 0.37, Q3: 1.17 kU_A_/L) will not be represented on ALEX^3^, since beech and hazel occur in the same regions as birch trees, and their PR-10 allergens were highly co-reactive with Bet v 1. As a third PR-10 allergen, Dau c 1 from carrot was removed from the array, because of low prevalence of IgE-reactivity and full coverage by other PR-10 proteins. In contrast, Aln g 1 from alder pollen showed up to 3.6-fold higher prevalence than Bet v 1 in defined regions of the world, e.g., Peru or Iran, and, therefore, will be kept on ALEX^3^. Finally, as a new PR-10 protein, Que a 1 from white oak was added, as this allergen is incompletely covered by Bet v 1 (see below, section on new allergens on ALEX^3^).

#### 2.2.3. Example of Non-Redundant Molecules of the Same Family: Ole e 1-like Proteins

**Figure 5:**
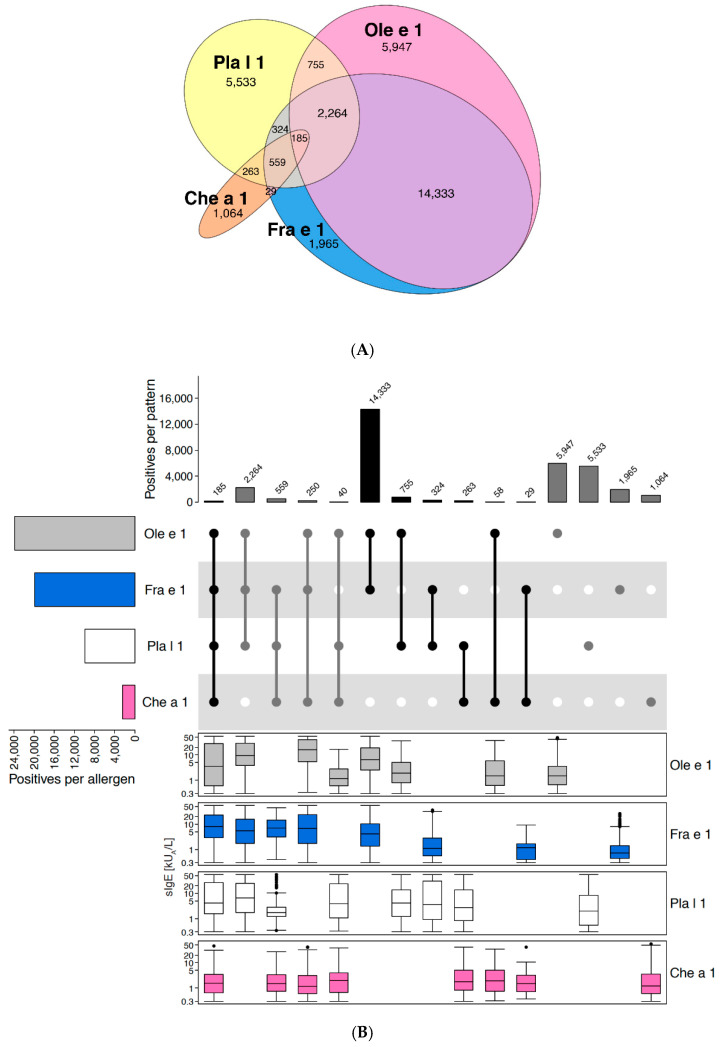
Ole e 1-like proteins. (**A**) Euler-plot of all samples positive to at least one of the Ole e 1-like allergens on ALEX^2^. Absolute numbers of cases are indicated for each respective segment. (**B**) Upset-plot (top), showing frequencies for different reactivity patterns to Ole e 1-like allergens. Numbers of cases for each individual allergen are indicated by bars on the left (“Positives per allergen”), and for each combination of IgE-reactivity by bars on top of the plot (“Positives per pattern”). Box-plots underneath each individual reactivity pattern show IgE-levels (kU_A_/L) to each of the abovementioned allergen preparations (y-axes in log_10_ scale). The combination matrix illustrates patterns of reactivity: a single dark (i.e., black or dark grey) dot represents samples monoreactive to the respective allergen on the left, two dark dots that are connected by a vertical line show samples with IgE-reactivity to the two corresponding allergens on the left side, etc. To facilitate readability, every second line is shown in light grey; white dots display absence of reactivity. Groups of reactivities to the same number of allergens are sorted from left (highest number of co-reactivities) to right (monoreactivities), each group either displayed by black or grey bars and dots, to facilitate identification of groups.

Of the Ole e 1-like protein family, four members are represented on ALEX^2^, i.e., Ole e 1 from olive pollen, Fra e 1 from ash tree pollen, Pla l 1 from ribwort plantain, and Che a 1 from goosefoot (also commonly designated “lamb’s quarters”). The Euler-plot in Figure 5A, which is based on 33,570 ALEX^2^ test results reactive to at least one of these four allergens, illustrates the limited co-reactivity between the latter. Even the more closely related allergens from olive tree and ash tree pollen showed only 64% co-reactivity among 26,361 cases positive to Ole e 1 and/or Fra e 1, while 11% were positive to Fra e 1 and negative to Ole e 1, and 25% vice versa. Co-reactivity with Pla l 1 and Che a 1 was even much lower, as demonstrated by 56% of monosensitisations for the former and 51% for the latter. This rather loose relation between these different Ole e 1-like proteins is also illustrated by the fact that average sIgE-levels to the two members from weeds (Che a 1 and Pla l 1) were only merely higher in samples showing co-reactivity with the corresponding allergens from olive and ash tree pollen (Figure 5B). This observation contrasts with results for other families, e.g., PR-10 allergens or storage proteins, and suggests an important role of epitopes that are specific for the individual Ole e 1-like allergens. Thus, all four allergens were kept on the ALEX^3^ array and were complemented by another member of this family, i.e., Sal k 5 from Russian thistle (*Salsola kali*, also designated “saltwort”).

#### 2.2.4. Example of Redundant Molecules of the Same Family: Profilins

**Figure 6:**
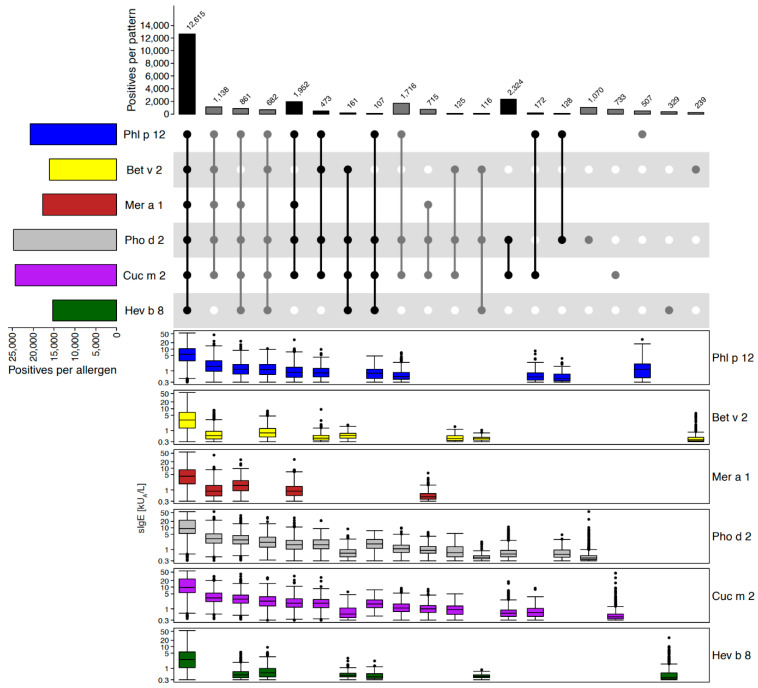
Profilins. Upset-plot (top), showing frequencies of reactivity patterns, and box-plots underneath each individual pattern showing IgE-levels (kU_A_/L, y-axes in log_10_ scale) to profilins on the ALEX^2^-test. Numbers of cases for each individual allergen are indicated by bars on the left (“Positives per allergen”), and for each combination of IgE-reactivity by bars on top of the Upset-plot (“Positives per pattern”). The combination matrix illustrates patterns of reactivity: a single dark (i.e., black or dark grey) dot represents samples monoreactive to the respective allergen on the left, two dark dots that are connected by a vertical line show samples with IgE-reactivity to the two corresponding allergens on the left side, etc. To facilitate readability, every second line is shown in light grey; white dots display absence of reactivity. Groups of reactivities to the same number of allergens are sorted from left (highest number of co-reactivities) to right (monoreactivities), each group either displayed by black or grey bars and dots, to facilitate identification of groups.

From the pan-allergen family of profilins, six different allergens are represented on ALEX^2^, four from pollen (Phl p 12, Bet v 2, Mer a 1 and Pho d 2), one from muskmelon (Cuc m 2) and one from latex (Hev b 8). In Figure 6, IgE-reactivity patterns and average sIgE-levels for each respective allergen and pattern are shown, based on a total of 27,200 ALEX^2^ test results positive to at least one profilin. The plot clearly demonstrates close-to-complete coverage of cases positive to profilin by Phl p 12 and Cuc m 2 (92.5%), while in those samples that were negative to these two profilins, average sIgE-levels were close to the cut-off of positivity (median sIgE levels: Bet v 2 = 0.38 kU_A_/L, Pho d 2 = 0.41 kU_A_/L, Hev b 8 = 0.4 kU_A_/L). Therefore, Phl p 12 and Cuc m 2 were identified as suitable surrogate markers for profilins and included on the ALEX^3^ array, while the other four profilins could be cleared.

### 2.3. Extracts Versus Allergen Families

#### Example: Grasses

**Figure 7:**
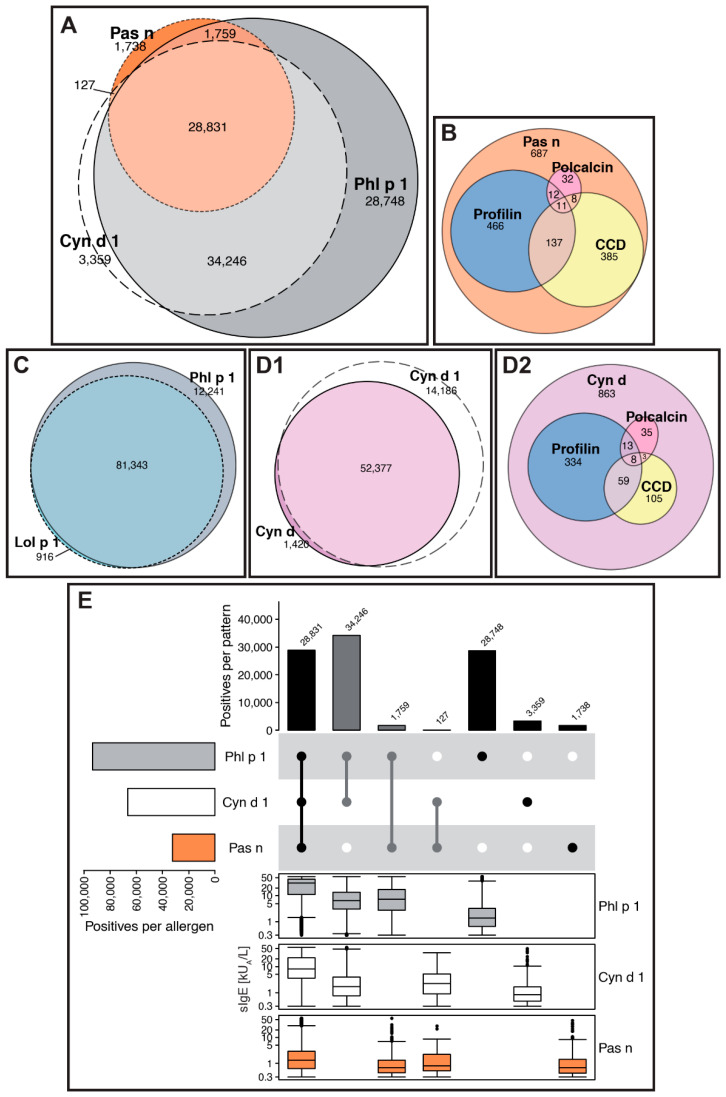
Grasses. (**A**–**D**) Euler-plots showing co-reactivity between (**A**) Phl p 1, Cyn d 1 and Bahia grass extract (Pas n), (**B**) Bahia grass extract and pan-allergens or CCDs, (**C**) Phl p 1 and Lol p 1, (**D1**) Bermuda grass extract (Cyn d) and Cyn d 1, and between (**D2**) Bermuda grass extract and pan-allergens (profilin, polcalcin) or CCDs. (**E**) Combined Upset-plot and box-plot, showing frequencies of positive ALEX^2^ test results for Phl p 1, Cyn d 1 and Pas n-extract (“Positives per allergen”) and of all patterns of co-reactivity thereof (“Positives per pattern”, upper part), and corresponding sIgE-levels (kU_A_/L, y-axes in log_10_ scale) for each individual pattern and component (bottom part). The combination matrix illustrates patterns of reactivity: a single dark (i.e., black or dark grey) dot represents samples monoreactive to the respective allergen on the left, two dark dots that are connected by a vertical line show samples with IgE-reactivity to the two corresponding allergens on the left side, etc. To facilitate readability, every second line is shown in light grey; white dots display absence of reactivity. Groups of reactivities to the same number of allergens are sorted from left (highest number of co-reactivities) to right (monoreactivities), each group either displayed by black or grey bars and dots, to facilitate identification of groups.

On ALEX^2^, both extracts and components from temperate (Timothy grass = *Phleum pratense*; Rye grass = *Lolium perenne*) and tropical grasses (Bermuda grass = *Cynodon dactylon*; Bahia grass = *Paspalum notatum*) are represented (Figure 7 and Figure S1). Phl p 1, Cyn d 1 and Bahia grass extract (Pas n), albeit concordant to a high degree, showed 29%, 3.4% and 1.8% of monosensitisations, respectively, in a total of 98,800 ALEX^2^ cases positive to at least one of those three allergen sources (Figure 7A). Likewise, from Figure 7E, it becomes apparent that Phl p 1 is the predominant group 1 grass pollen allergen which, however, is likely to be biased by most of the data originating from temperate climate zones. Those observations are suggestive of specific epitopes on individual group 1 grass pollen allergens (Figure S1, sIgE-levels for monosensitised samples). However, average sIgE-levels were highest in sera with IgE-positivity to Phl p 1, Cyn d 1 and Pas n-extract, which is even more pronounced when including additional grass pollen allergens present on ALEX^2^ (Figure S1), which supports the previously reported strong cross-reactivity between those grasses [23].

For Bahia grass (Pas n) extract, monosensitised samples from Figure 7A were analysed for IgE-reactivity to pan-allergens (profilins or polcalcins) and CCDs (Figure 7B). Samples exclusively positive to Pas n-extract showed merely lower average sIgE-levels (median: 0.56 kU_A_/L, Q1: 0.39 kU_A_/L, Q3: 1.16 kU_A_/L) as compared to results co-reactive to other grass pollen allergens (Figure 7E, Figure S1, e.g., columns 14 or 20). This finding probably suggests individual epitopes on the major group 1 grass pollen allergen contained in the Pas n-extract. Therefore, to guarantee high sensitivity of detection of IgE-sensitisation to, both, temperate and tropical grasses, Phl p 1, Cyn d 1 and Pas n-extract were included in the ALEX^3^ allergen list. Because of a varying degree of cross-reactivity with other group 1 grass pollen allergens reported in the literature, Zea m 1 from maize pollen was included as an additional beta-expansin [24].

In contrast, Lol p 1 from Rye grass was cleared from the array due to close-to-complete coverage by Phl p 1 (Figure 7C) and comparably low average sIgE values of Lol p 1-monosensitsed samples (median: 0.52 kU_A_/L, Q1: 0.39 kU_A_/L, Q3: 0.92 kU_A_/L, also refer to Figure S1). For Bermuda grass, the extract was removed from the array, as the by far biggest share was covered by Cyn d 1 on ALEX^2^ (Figure 7D1), i.e., 98% of Bermuda-grass positive samples (n = 67,983). The remaining Cyn d-monosensitised samples from Figure 7D1 which were negative to pan-allergens (profilins, polcalcins) and CCDs (n = 863, Figure 7D2) exhibited low sIgE-levels to Cyn d-extract (median: 0.57 kU_A_/L, Q1: 0.38 kU_A_/L, Q3: 1.09 kU_A_/L). Therefore, and due to the coverage of Bermuda grass by the major component, Cyn d 1, the Bermuda grass extract was removed from the array.

### 2.4. Extracts and Allergens with Low Prevalence of Sensitisation

Those extracts that showed a low prevalence of sensitisation (i.e., <1%) and median sIgE-levels close to the cut-off of ALEX^2^ (0.3 kU_A_/L) were not included in the ALEX^3^ array. Examples are oregano (*Origanum vulgare*, 0.26% positivity), parsley (*Petroselinum crispum*, 0.44%), caraway (*Carum carvi*, 0.61%) or rice (*Oryza sativa*, 0.83%). Furthermore, the extracts from common mussel (*Mytilus edulis*) or oyster (*Ostrea edulis*) were excluded since they were prone to yield higher background levels. The lower part of Table S1 provides an overview of all allergens and extracts that were not included in the ALEX^3^ array.

### 2.5. New Allergens on ALEX^3^

While for the assessment of the relevance of allergens and extracts on ALEX^2,^ we could use a big set of real-world ALEX^2^ data, the decision on which additional allergens to include on the updated array was based on scientific literature and on our own studies conducted in collaboration with research groups worldwide. As a result, a total of 52 new allergens from animal-sourced food, plant-based food, respiratory allergen sources or venom allergens were added, including markers for two additional pan-allergen families.

The sugar-epitope Galactose-α-1,3-galactose (commonly designated “Alpha-gal”), as an example, is clearly distinct from clinically irrelevant CCDs and indicates sensitisation to red meat which can even lead to delayed anaphylaxis, as was shown in numerous clinical trials [25].

The haemocyanin from white shrimp (*Litopenaeus vannamei*), Lit v 7, is an illustrative example of differences between regional exposomes that are related to distinct habits. Haemocyanin is a protein contained at high concentrations in the haemolymph of invertebrates, including shrimp [26,27]. In countries where the cephalothorax, i.e., the inner part of the head and thorax of the shrimp, is commonly consumed, such as in Thailand or Spain, but also in industries processing shrimp, people are exposed to high levels of that allergenic protein via the gastrointestinal or the respiratory route and, hence, might become sensitised. To achieve improved sensitivity for the detection of shrimp-sensitisation, haemocyanin from white shrimp (Lit v 7), the shrimp species with the highest share of world production [28], was included on ALEX^3^. In addition, Mac r 1 and 2, i.e., tropomyosin and arginine kinase from the giant freshwater prawn (*Macrobrachium rosenbergii*) were added to the ALEX^3^ array. Annual production and, hence, consumption of this prawn species is constantly increasing [28]. Importantly, these two allergens differ in amino acid sequences compared to the corresponding allergens from black tiger prawn (*Penaeus monodon*) and from white shrimp (*Litopenaeus vannamei*) and, therefore, add additional coverage of seafood allergens to the ALEX^3^-test.

As another important animal source food allergen, Gal d 7, the myosin light chain 1 from chicken meat was added to the array [29]. In contrast to Gal d 5 (chicken serum albumin), which is a marker for secondary allergy to chicken meat after primary sensitisation to egg yolk or serum albumin carried by bird feathers (bird-egg or egg-bird syndrome), Gal d 7 is a marker for primary allergy to chicken meat.

The list of plant-based food allergens was complemented, among others, by two allergens from banana, Mus a 2 (class I chitinase) and Mus a 5 (β-1,3-glucanase), and one from mango, Man i 1 (class IV chitinase). Allergy to these fruits, particularly prevalent in Asian populations [30,31,32], can cause severe clinical manifestations [33,34]. In contrast, wheat, as a member of the “big eight” (recently grown to the “big nine” after including sesame), represents one of the most frequent causes of food allergies worldwide [35]. Coverage of wheat allergens will be improved on ALEX^3^ due to the addition of Tri a 36 (low molecular weight glutenin) [36] and Tri a 37 (α-purothionin) [37,38,39] to the wheat allergen panel.

In addition to new food allergens, further respiratory allergens were added to ALEX^3^. The birch pollen allergen Bet v 7, for example, serves as a surrogate marker for the ubiquitous pan-allergen family of cyclophilins [40] and will complement the detection of sensitisation to two other main families of pan-allergens, i.e., profilins and polcalcins. Likewise, among the two new fungal allergens of ALEX^3^, Asp f 8 and Mala s 13, the latter represents a surrogate marker for the fungal pan-allergen family of thioredoxins [41].

The major allergen from white oak (*Quercus alba*), Que a 1, was included on ALEX^3^ because of the limited cross-reactivity with the corresponding PR-10 protein from silver birch pollen, Bet v 1 [42], which is the main marker for sensitisation to this allergen family on ALEX^2^ (Figure 4). Que a 1 will provide improved coverage of allergic sensitisation to different oak species which are particularly prevalent in North America, parts of Asia (e.g., South Korea) or on the Iberian Peninsula.

Finally, the panel of venom allergens was improved by adding Api m 2 from honeybee venom to the allergen list. Since CCD-specific IgE is inhibited by default on the ALEX-test, the CCD-bearing Api m 2 can be employed as an additional marker for the differentiation between allergic sensitisation to honeybee venom and wasp venom [26]. A second improvement was achieved by replacing the extract of the venom from the white-faced hornet (*Dolichovespula maculata*) with the components Dol m 2 and Dol m 5 to increase sensitivity for the detection of sensitisation to that species [43].

### 2.6. Summary of Changes from ALEX^2^ to ALEX^3^

In total, the number of allergen molecules was increased from 178 on ALEX^2^ to 218 on ALEX^3^, increasing the number of represented allergen families from 74 to 80. In parallel, the number of extracts tapered from 117 to 82 (Table S1). In summary, 12 allergens and 36 extracts were removed from the array, while 52 new allergen molecules and one extract were included on ALEX^3^ (the complete allergen list is provided in Table S2). After the removal of allergens and extracts of negligible clinical relevance, the total number of allergen sources covered by ALEX^3^ amounts to 145. Two allergens already comprised on ALEX^2^, Mac i 1.0101 from macadamia nut and Pap s 1.0101 from poppy seed, were retained on ALEX^3^ but assigned to the family of α-hairpinins, instead of the 2S albumin family [44].

## 3. Discussion

The primary goal of the ongoing stepwise evolution of the ALEX macroarray is to maximise the diagnostic sensitivity of the test. One cornerstone to achieve this goal is the careful selection of those allergens that ensure coverage of a broad range of clinically relevant allergen sources and which offer the possibility to differentiate between genuine and cross-sensitisations. As an example, using only Der p 1 and Der p 2 to detect sensitisation to the European house dust mite, *Dermatophagoides pteronyssinus*, while omitting Der p 23, would result in false negative results in around 10% of mite-sensitised subjects who are monosensitised to Der p 23 (Figure S2), a clinically relevant mite-specific marker allergen [45].

Selection of allergens for ALEX^3^ was largely based (1.) on peer-reviewed scientific literature, (2.) on results from clinical studies conducted in collaboration with partners in different countries, and (3.) on analysis of a large dataset of almost 400,000 real-world ALEX^2^ test results. From the latter analyses, we obtained detailed patterns of reactivity for allergen families with restricted (e.g., storage protein families, Figure 3), moderate (e.g., PR-10 proteins, Figure 4) or strong co-reactivity (e.g., profilins, Figure 6). Those results provided the rationale for the decision of which allergens ought to be kept on or removed from the array (Table S1). For families with a high degree of observed co-reactivity, e.g., profilins and polcalcins, we retained two members per family as surrogate markers for sensitisation to the family as a whole. Of note, such a surrogate marker can be used worldwide, irrespective of the prevalence of the respective allergen source. IgE-reactivity to Phl p 12 from timothy grass (a temperate grass), for example, indicates sensitisation to the profilin family, even in regions, where timothy grass does not exist.

This was different from allergen families with restricted co-reactivity, e.g., storage proteins, nsLTPs, etc. For the sensitive detection of IgE-sensitisation to such families, a comprehensive array of allergens is vital, particularly for allergens that convey a high risk of severe clinical reactivity.

The second aim of the design of ALEX^3^ was to incrementally substitute extracts with allergen molecules, to overcome substantial technical constraints associated with extracts (see introduction), and to further augment the resolution of the test [46,47]. For those allergen sources of which both extracts and components are represented on ALEX^2^, we analysed 400,000 ALEX^2^ test results for co-reactivity between the two. Many results showing positivity to the extract, while being negative to the corresponding component, could be attributed to IgE-reactivity to pan-allergens (profilins, polcalcins) or CCDs. Although a CCD-blocker is included by default in the ALEX sample diluent, around 1.5% of all samples contain high levels of CCD-specific IgE, leading to incomplete inhibition by the blocker. Examples of such analyses are provided in Figure 1, Figure 2 and Figure 7. Those remaining cases that had tested negative to pan-allergens and CCDs as well can be explained by IgE-reactivity to a different isoform of the same allergen, or to distinct specific or cross-reactive allergens in the extract that are not yet represented on the ALEX-test. In total, we found high coverage of allergen sources by the selected allergens on ALEX^2^ which allowed for the decision to omit many extracts on ALEX^3^, as summarised in Table S1. However, for allergen sources with a substantial proportion of IgE-monoreactivity to the extract that could not be attributed to pan-allergens or CCDs, the respective extract was kept on ALEX^3^, even in case of low average sIgE-levels.

The opposite scenario, i.e., a positive result for the component and no reactivity with the extract, is mostly due to the fact that a spot on the array that contains only one defined allergen molecule comprises more copies of that molecule than the corresponding extract, which is a complex mixture of allergenic and non-allergenic constituents. The concentration of an allergen in the extract is always lower than in a preparation of the same allergen as purified natural or recombinant protein. Hence, in samples with a low sIgE-level to a particular allergen, the “pure” molecule is more sensitive to detect IgE-reactivity than the extract.

This discrepancy might be even exacerbated by the fact that each allergen source comprises allergen molecules that belong to different protein families with distinct physicochemical properties. Depending on the extraction protocol applied, particular allergens are enriched from the raw material while others are hardly solubilised. One example where this limitation causes substantial variation between different extraction protocols is wheat. Therefore, allergen extracts are hardly ever complete nor comparable in terms of their allergen composition. One strategy to tackle this limitation is the spiking of extracts with purified allergen molecules that are underrepresented [48]. This is being done for selected extracts on ALEX^2^, however, from the perspective of an assay developer, it does not make sense to include two or more spots on an array that convey the same information, such as an extract and a component of the same source that show concordant reactivities. Ideally, an extract should complement but not duplicate results from corresponding components. In this respect, such a complementary extract ought to be produced by applying an extraction procedure that enriches other allergens than those already present as purified components on the test. Hence, for ALEX^3^, extracts will no longer be spiked with purified allergens. However, from the user’s perspective, under the (mostly unmet) assumption that extracts usually contain all clinically relevant allergens at sufficient concentrations, a serum testing positive for the component but negative for the corresponding extract might raise the concern that the extract is faulty. Therefore, transparency from the manufacturer’s side with respect to the used allergen preparations is vital to avoid such misconceptions.

Finally, those extracts which had a low prevalence (i.e., <1%), combined with questionable clinical relevance will not be included on ALEX^3^ due to negligible diagnostic significance (Table S1).

The third major aim of the development of ALEX^3^ was to ensure broad coverage of exposomes [16,49] on a global scale. Exposomes from various regions of the world might show concordance with respect to ubiquitous and hardly variable allergen sources, such as house dust mites, grasses or furry animals. However, differences in climatic conditions and overall lifestyle might lead to peculiarities in the pool of allergen molecules people are exposed to and, therefore, in their IgE-reactivity profiles. Climatic conditions determine the repertoire of plants and animals thriving in the respective area [50,51], the onset and duration of pollen seasons, as well as the number of pollen shed [52,53], or allergen content per pollen grain [54,55]. Lifestyle differences are reflected by specific habits and traditions, e.g., preparing dishes, keeping particular species as domestic animals, (abundant) use of detergents or disinfectants, etc. Together with other factors, such as the predominance of industry and individual motor car traffic [56], leading to high particle density in ambient air, and many others, those parameters determine the composition of the allergenic environment, allergen load (season- and concentration-wise) and immunological effects of exposure. Thus, for a globally used multiplex IgE-test, it is vital to encompass region-specific as well as ubiquitous allergen molecules. Therefore, within the scope of worldwide research collaborations, the ALEX-test is constantly scrutinised regarding its diagnostic sensitivity to detect region-specific allergic sensitisations. In these studies, the clinical reactivity of the patient defines the ground truth, since, due to the lack of standard reagents for sIgE-testing, none of the existing tests to detect allergic sensitisation can be assigned the label of a “gold standard” and, hence, of a valid reference [57]. Learnings from those collaborations and from the scientific literature were translated into the new version of the ALEX-test as illustrated by the addition of 52 new allergen molecules and one additional extract (for details please refer to the results section). Once ALEX^3^ is launched, results from continuous surveillance of each allergen’s and extract’s performance will be used for the design of the subsequent version of the test, i.e., ALEX^4^.

In conclusion, this paper demonstrates how a large compilation of real-world data of the ALEX^2^ macroarray is used for the targeted design of a follow-up version of the test, clearing diagnostically irrelevant allergens, incrementally substituting extracts by allergen molecules and including new allergens with the aim to further increase diagnostic sensitivity of the test.

One limitation of our study is that most data (around 70%) originate from European countries. However, as data from regions outside Europe continue to accumulate, we expect to observe a shift in prevalences for distinct allergens that are more prevalent in regions that are currently underrepresented in the existing dataset. Therefore, due to the large number of data, such potential bias can be compensated in future analyses by random selection of equal numbers of test results from distinct regions.

## 4. Materials and Methods

### 4.1. ALEX^2^ Macroarray

The ALEX^2^ (Allergy Xplorer) macroarray (MacroArray Diagnostics, Vienna, Austria) is a diagnostic test for the simultaneous detection of specific IgE to 178 different allergen molecules and 117 extracts, together with total IgE [58]. A literature review providing a comprehensive overview of more than 50 clinical studies where ALEX^2^ was used is in preparation [59].

On the ALEX^2^-test, each respective allergen preparation is coupled to nanoparticles prior to spotting onto a nitrocellulose membrane. Before incubation on the test for 2h at room temperature (RT), sera are diluted 1:5 using a sample diluent composed of Tris-buffer, Tween and Na-azide, that contains HRP as a CCD-blocker. For the detection of CCD-specific IgE that could not be fully blocked by the inhibitor, mostly because of excessively elevated IgE-levels, human lactoferrin (Hom s LF) that carries CCD-sugar moieties, is comprised on the test as a CCD-marker. For colorimetric detection of IgE bound to the solid phase, the array is incubated for 30 min with an IgE-specific detection antibody that is conjugated with alkaline phosphatase. After incubation with an AP-substrate solution for 8 min, followed by the addition of a stop solution, a picture of the dried array is captured by a charge-coupled device camera. Analysis of the array is done by RAPTOR software (MacroArray Diagnostics, Vienna, Austria), converting the intensity of the colour reaction per spot into kU_A_/L for specific IgE, and into kU/L for total IgE, by using a five-point calibration curve. Between January 2020 and June 2023, when data for the present study were generated, versions 1.1.0.43 to V1.12.0 of the RAPTOR software were in use. Details of the protocol and composition of reagents are provided by the manufacturer in the instructions for use (www.macroarraydx.com, accessed on 3rd of February 2025).

Processing of samples can be done either manually when using the Image Xplorer system for image acquisition, or fully automated by the MAX9k or MAX45k systems (MacroArray Diagnostics). Most ALEX^2^ data analysed for the present study were generated by one of the two automated systems.

### 4.2. ALEX^2^ Dataset

Results from ALEX-tests performed in more than 90 countries worldwide (as of December 2024) are stored on a GDPR-compliant server. By accepting the terms and conditions, users consent to the use of the data for continuous postmarket surveillance and for scientific purposes. By mid-2023, when ALEX^3^ was designed, more than 396,000 ALEX^2^ test results were available. By the end of 2024, this number has more than doubled, amounting to 895,000 test results. All plots shown in this paper are based on data that were generated between January 2020 and mid-2023 and which were used for the development of the ALEX^3^-array. Depending on the respective analysis, test results that exceeded the test’s cut-off of (i.e., ≥0.3 kU_A_/L) for specified allergens and/or extracts were selected from the complete dataset.

For the present paper, a retrospective analysis of the specified ALEX^2^ dataset was done. The data represent results from routine testing of sera from subjects who had a consultation at various allergy departments, private practices and diagnostic laboratories across the world, supposedly because of the clinical suspicion of allergies. Since no additional blood taking, data collection or other measures affecting patients were carried out, no approval by an ethics committee was required. The type of analysis and presentation of the data precludes the identification of individual patients, clinical departments or laboratories.

### 4.3. Data Analysis

To assess the coverage of an allergen extract by one or more allergens of the same allergen source and by pan-allergens (profilins, polcalcins) or CCDs (represented on ALEX^2^ by the CCD-marker Hom s LF), Euler-plots were generated and average sIgE-levels (medians, Q1 and Q3) were calculated for the different segments. For more complex visualisations involving more than four allergens/extracts, Upset-plots were generated, combined with box-plots to convey information on average sIgE-levels for each individual allergen/extract for the respective combination of IgE-reactivities. The latter type of plot was also employed to visualise IgE-reactivity patterns to different members of the same allergen family represented on ALEX^2^.

Calculation of sIgE-levels and plotting of data was done by using RStudio software V2024.12.1 (Posit Software, PBC, Boston, MA, USA) [60]. Combined Upset-plots and box-plots were generated using the Complex heatmap package [61].

Testing for statistical significance was not done, since for large numbers of samples, as was true for the present analyses, even minuscule differences between groups often reach statistical significance although they are not relevant for diagnostic purposes. In general, the data shown in the present paper represented one main foundation for our decisions on the design of the ALEX^3^ allergen panel. However, the final decision to keep or remove particular allergens/extracts was made case by case, considering clinical relevance (high-risk vs. low-risk allergens), frequencies of reactivity, degree of co-reactivity, etc.

## Data Availability

The original data presented in this study are the property of MacroArray Diagnostics. Further inquiries can be directed to the corresponding author.

## Supplementary Materials

The supporting information can be downloaded at: https://www.mdpi.com/article/10.3390/ijms26094249/s1.
