# Supplementary material for: Big Data-Driven Evolution of a Diagnostic Multiplex IgE-Test: Enhancing Accuracy and Efficacy in Allergy Diagnostics
Source: Int J Mol Sci. 2025 Apr 29;26(9):4249. doi: 10.3390/ijms26094249 (PMC12072476; doi:10.3390/ijms26094249)
Supplement: Supplementary file 1 [file ijms-26-04249-s001.zip › ijms-3531690-supplementary.pdf]

SUPPLEMENTARY MATERIAL

Big data-driven evolution of a diagnostic multiplex IgE-test: Enhancing accuracy and efficacy in allergy diagnostics

Christian Lupinek[1], Peter Forstenlechner[1], Anna Ringauf[1], Raffaela Campana[1], Artan Salihu[1], Martina Aumayr[1], Irene Mittermann[1]

[1]MacroArray Diagnostics GmbH, Lemboeckgasse 59/4, 1230 Vienna, Austria

Corresponding author:
Christian Lupinek
MacroArray Diagnostics GmbH
Lemboeckgasse 59/4
1230 Vienna, Austria
e-mail: lupinek@macroarraydx.com

Supplementary tables and figures

Table S1. ALEX^2 versus ALEX^3: New and discontinued allergens and extracts

<table>
<tr><th colspan="4">New allergen molecules and extracts on ALEX^3</th></tr>
<tr><th>Allergen/Extract</th><th>Allergen family</th><th>Allergen source</th><th>Relevance</th></tr>
<tr><td colspan="4">Pollen</td></tr>
<tr><td>rQue a 1</td><td>PR-10</td><td>Oak</td><td>Limited cross-reactivity to Bet v 1</td></tr>
<tr><td>rBet v 7</td><td>Cyclophilin</td><td>Birch</td><td>Surrogate-marker for pan-allergen family</td></tr>
<tr><td>nOle e 7</td><td>nsLTP</td><td>Olive</td><td>Important marker in Southern Europe</td></tr>
<tr><td>nZea m 1</td><td>β-expansin</td><td>Maize, pollen</td><td>Limited cross-reactivity with Phl p 1</td></tr>
<tr><td>rSal k 5</td><td>Ole e 1 family</td><td>Russian thistle</td><td>Improved coverage</td></tr>
<tr><td colspan="4">Mites, insects and insect venom</td></tr>
<tr><td>rDer f 15</td><td>Chitinase</td><td rowspan="2">American house dust mite</td><td rowspan="3">Improved coverage</td></tr>
<tr><td>rDer f 18</td><td>Chitinase-like protein</td></tr>
<tr><td>rBlo t 2</td><td>NPC2 family</td><td>Blomia tropicalis</td></tr>
<tr><td>rTyr p 10</td><td>Tropomyosin</td><td>Tyrophagus putrescentiae</td><td>Surrogate-marker for pan-allergen family</td></tr>
<tr><td>rPer a 6</td><td>Troponin C</td><td>American cockroach</td><td rowspan="2">Improved coverage</td></tr>
<tr><td>rApi m 2</td><td rowspan="2">Hyaluronidase</td><td>Honeybee</td></tr>
<tr><td>rDol m 2</td><td rowspan="2">Bald-faced hornet</td><td rowspan="2">Replacement for extract</td></tr>
<tr><td>rDol m 5</td><td>Antigen 5</td></tr>
<tr><td colspan="4">Furry animals</td></tr>
<tr><td>rMes a 1</td><td rowspan="2">Lipocalin</td><td>Golden hamster</td><td>Low cross-reactivity with other lipocalins</td></tr>
<tr><td>rRat n 1</td><td>Rat</td><td>Replacement for extract</td></tr>
<tr><td colspan="4">Fungi</td></tr>
<tr><td>rAsp f 8</td><td>Ribosomal protein 2</td><td>Aspergillus fumigatus</td><td rowspan="2">Improved coverage</td></tr>
<tr><td>rMala s 13</td><td>Thioredoxin</td><td>Malassezia sympodialis</td></tr>
<tr><td colspan="4">Plant-based food</td></tr>
<tr><td>rPers a 1</td><td rowspan="2">Class I chitinase</td><td>Avocado</td><td>Marker for latex-fruit syndrome</td></tr>
<tr><td>rMus a 2</td><td rowspan="2">Banana</td><td>Marker for latex-fruit syndrome; Replacement for extract</td></tr>
<tr><td>rMus a 5</td><td>β-1,3-glucanase</td><td rowspan="2">Replacement for extract</td></tr>
<tr><td>rMan i 1</td><td>Class IV chitinase</td><td>Mango</td></tr>
<tr><td>rPru p 7</td><td>Gibberellin-regulated protein</td><td>Peach</td><td>Improved coverage</td></tr>
<tr><td>rTri a 36</td><td>LMW glutenin</td><td rowspan="2">Wheat</td><td>Improved risk assessment</td></tr>
<tr><td>rTri a 37</td><td>α-purothionin</td><td>Anaphylaxis marker</td></tr>
<tr><td>rCar i 1</td><td>2S albumin</td><td>Pecan</td><td>Improved risk assessment</td></tr>
</table>

New allergen molecules and extracts on ALEX^{3}			
**Allergen/Extract**	**Allergen family**	**Allergen source**	**Relevance**
rPin p 1	2S albumin	Pine nut	New allergen source
nMac i 1.0101 (28-76)	α-hairpinin	Macadamia	Assigned to new allergen family, improved risk assessment
nPap s 1.0101 (27-846)		Poppy seed	
nAna o 1	7/8S globulin	Cashew	Improved risk assessment, replacement for extract
rCar i 2 (256-386)		Pecan	Improved risk assessment
rLen c 1		Lentil	Replacement for extract
nCoc n 1		Coconut	New allergen source
rPis s 1		Pea	Replacement for extract
rPis s 2			
rCar i 4	11S globulin	Pecan	Improved risk assessment
nPru du 6		Almond	
Pin p	(Extract)	Pine nut	New allergen source
rHel a 3	nsLTP	Sunflower seed	Replacement for extract, improved risk assessment
rPru av 3		Cherry	
rLen c 3		Lentil	
rPis s 3		Pea	
rFra a 3		Strawberry	Replacement for Fra 1 + 3 mix
rAra h 18	Cyclophilin	Peanut	Surrogate-marker for pan-allergen family
rApi g 7	Plant defensin	Celery	Marker for mugwort-celery syndrome
Animal source food			
rBos d 9	α-S1 casein	Cow's milk	Improved coverage and resolution
rBos d 10	α-S2 casein		
rBos d 11	β-casein		
nBos d 12	κ-casein		
rGal d 7	Myosin light chain	Chicken meat	Major chicken meat allergen
rCyp c 2	β-enolase	Carp	Replacement for Gad m 2
nSal s 6	Collagen	Salmon	Improved sensitivity
rMac r 1	Tropomyosin	Giant freshwater prawn	New allergen source
rMac r 2	Arginine kinase		
nLit v 7	Haemocyanin	White shrimp	Improved coverage
Galactose-α-1,3-galactose	α-gal	Red meat	

Discontinued allergen molecules and extracts			
**Allergen/Extract**	**Allergen family**	**Allergen source**	**Rationale**
Pollen			
rFag s 1	PR-10	Beech	Covered by other PR-10 proteins
rCor a 1.0103		Hazel pollen	

Discontinued allergen molecules and extracts			
**Allergen/Extract**	**Allergen family**	**Allergen source**	**Rationale**
Cor a pollen	(Extracts)	Hazel pollen	Coverage by allergen molecules
Fra e		Ash	
Pop n		Cottonwood	Negligible diagnostic relevance
Ulm c		Elm	
Mor r		Mulberry tree	
rLol p 1	Beta-expansin	Perennial ryegrass	Covered by Phl p 1
Cyn d	(Extracts)	Bermuda grass	Covered by Cyn d 1
Urt d		Nettle	Negligible diagnostic relevance
Pla l		Ribwort	Covered by Pla l 1
rPho d 2	Profilin	Date palm	Covered by other profilins
rBet v 2		Silver birch	
rMer a 1		Annual mercury	
Mites and insect venom			
rDer p 11	Myosin heavy chain	European house dust mite	Negligible diagnostic relevance
Ves v	(Extracts)	Common wasp venom	Covered by Ves v 1 & 5
Dol spp.		Long-headed wasp venom	Covered by Dol m 2 & 5
Furry animals			
Rat n	(Extracts)	Rat	Replaced by Rat n 1
Ovi a epithelium		Sheep	Covered by other epithelia extracts
Latex			
rHev b 8	Profilin	Latex	Covered by other profilins
Plant-based food			
Ana o	(Extracts)	Cashew	Replaced by Ana o 1-3
Tri fo		Fenugreek seed	Negligible diagnostic relevance
Ory s		Rice	
Pha v		Green bean	
Len c		Lentil	Replaced by Len c 1 & 3
Pis s		Pea	Replaced by Pis s 1-3
Pim a		Anise	Negligible diagnostic relevance
Car c		Caraway	
Ori v		Oregano	
Cap a		Bell pepper	
Pet c		Parsley	
rMal d 2	TLP	Apple	Covered by Act d 2
Mus a	(Extracts)	Banana	Replaced by Mus a 2 & 5
Vac m		Blueberry	Negligible diagnostic relevance
Pru av		Cherry	Replaced by Pru av 3

Discontinued allergen molecules and extracts			
**Allergen/Extract**	**Allergen family**	**Allergen source**	**Rationale**
Man i	(Extracts)	Mango	Replaced by Man i 1
Cit s		Orange	Negligible diagnostic relevance
rFra a 1 + 3	PR-10 & LTP-mix	Strawberry	Replaced by Fra a 3
Dau c	(Extract)	Carrot	Covered by other allergen molecules
rDau c 1	PR-10	Carrot	Covered by other PR-10 allergens
Sac c	(Extract)	Baker's yeast	Negligible diagnostic relevance
Animal source food			
Gad m	(Extract)	Atlantic cod	Negligible diagnostic relevance
nGad m 2 + 3	β-enolase & aldolase-mix	Atlantic cod	Replaced by Cyp c 2
Myt e	(Extracts)	Common mussel	Negligible diagnostic relevance
Ost e		Oyster	
Pec spp.		Scallop	
Thu a		Tuna	
Sus d meat		Pork	

Table S2. Complete list of allergen molecules and extracts comprised on ALEX[3].

Allergen/Extract	Allergen family	Allergen source
Grass pollen		
Pas n	(Extract)	Bahia grass
Cyn d 1	β-expansin	Bermuda grass
Phr c	(Extract)	Common reed
Zea m 1	β-expansin	Maize, pollen
Sec c_pollen	(Extract)	Rye, pollen
Phl p 1	β-expansin	Timothy grass
Phl p 2	Expansin	
Phl p 5.0101	Grass group 5/6	
Phl p 6		
Phl p 7	Polcalcin	
Phl p 12	Profilin	
Tree pollen		
Aca m	(Extract)	Acacia
Aln g 1	PR-10	Alder
Aln g 4	Polcalcin	
Fra e 1	Ole e 1 family	Ash
Bet v 1	PR-10	Birch
Bet v 6	Isoflavone reductase	

Allergen/Extract	Allergen family	Allergen source
Bet v 7	Cyclophilin	Birch
Cup a 1	Pectate lyase	Arizona cypress
Cup s	(Extracts)	Cypress
Jun a		Mountain cedar
Que a 1	PR-10	Oak
Ole e 1	Ole e 1 family	Olive
Ole e 7	nsLTP	
Ole e 9	β-1,3-glucanase	
Bro pa	(Extract)	Paper mulberry
Pla a 1	Plant invertase inhibitor	London plane tree
Pla a 2	Polygalacturonase	
Pla a 3	nsLTP	
Cry j 1	Pectate lyase	Sugi
Ail a	(Extracts)	Tree of heaven
Jug r_pollen		Walnut
Weed pollen		
Ama r	(Extracts)	Pigweed
Can s		Hemp
Can s 3	nsLTP	
Che a	(Extract)	Lamb's quarter
Che a 1	Ole e 1 family	
Art v	(Extract)	Mugwort
Art v 1	Plant defensin	
Art v 3	nsLTP	
Amb a	(Extract)	Ragweed
Amb a 1	Pectate lyase	
Amb a 4	Plant defensin	
Pla l 1	Ole e 1 family	Ribwort
Sal k	(Extract)	Russian thistle
Sal k 1	Pectin methylesterase	
Sal k 5	Ole e 1 family	
Par j	(Extract)	Wall pellitory
Par j 2	nsLTP	
Mites		
Aca s	(Extract)	Acarus siro
Der f 1	Cysteine protease	American house dust mite
Der f 2	NPC2 family	
Der f 15	Chitinase	
Der f 18	Chitinase-like protein	
Blo t 2	NPC2 family	Blomia tropicalis
Blo t 5	Mite group 5/21	
Blo t 10	Tropomyosin	
Blo t 21	Mite group 5/21	
Der p 1	Cysteine protease	European house dust mite
Der p 2	NPC2 family	
Der p 5	Mite group 5/21	

Allergen/Extract	Allergen family	Allergen source
Der p 7	Mite group 7	European house dust mite
Der p 10	Tropomyosin	
Der p 20	Arginine kinase	
Der p 21	Mite group 5/21	
Der p 23	Peritrophin-like protein domain	
Gly d 2	NPC2 family	Glycyphagus domesticus
Lep d 2		Lepidoglyphus destructor
Tyr p	(Extract)	Tyrophagus putrescentiae
Tyr p 2	NPC2 family	
Tyr p 10	Tropomyosin	
Animal dander and epithelia		
Fel d 1	Uteroglobin	Cat
Fel d 2	Serum albumin	
Fel d 4	Lipocalin	
Fel d 7		
Bos d 2		Cattle
Phod s 1		Djungarian hamster
Can f 1		Dog
Can f 2		
Can f 3	Serum albumin	
Can f 4	Lipocalin	
Can f 6		
Can f Fel d 1 like	Uteroglobin	
Can f_male urine	(Extracts)	Male dog urine (incl. Can f 5)
Cap h_epithelia		Goat, epithelia
Mes a 1	Lipocalin	Golden hamster
Cav p 1		Guinea pig
Equ c 1		Horse
Equ c 3	Serum albumin	
Equ c 4	Latherin	
Mus m 1	Lipocalin	Mouse
Sus d_epithelia	(Extract)	Pig, epithelia
Ory c 1	Lipocalin	Rabbit
Ory c 2		
Ory c 3	Uteroglobin	
Rat n 1	Lipocalin	Rat
Fungi		
Alt a 1	Alt a 1 family	*Alternaria alternata*
Alt a 6	Enolase	
Asp f 1	Mitogillin family	*Aspergillus fumigatus*
Asp f 3	Peroxysomal protein	
Asp f 4	Unknown	
Asp f 6	Mn superoxide dismutase	
Asp f 8	Ribosomal protein 2	
Cla h	(Extract)	*Cladosporium herbarum*
Cla h 8	Mannitol dehydrogenase	

Allergen/Extract	Allergen family	Allergen source
Mala s 5	Unknown	*Malassezia sympodialis*
Mala s 6	Cyclophilin	
Mala s 11	Mn superoxide dismutase	
Mala s 13	Thioredoxin	
Pen ch	(Extract)	*Penicillium chrysogenum*
	Insects and venoms	
Per a	(Extract)	American cockroach
Per a 6	Troponin C	
Per a 7	Tropomyosin	
Ves v 1	Phospholipase A1	Common wasp
Ves v 5	Antigen 5	
Sol spp.	(Extract)	Fire ant
Bla g 1	Nitrile specifier	German cockroach
Bla g 2	Aspartic protease	
Bla g 4	Calycin	
Bla g 5	Glutathion S-transferase	
Bla g 9	Arginine kinase	
Api m	(Extract)	Honeybee
Api m 1	Phospholipase A2	
Api m 2	Hyaluronidase	
Api m 10	Icarapin variant 2	
Dol m 2	Hyaluronidase	Bald-faced hornet
Dol m 5	Antigen 5	
Pol d	(Extract)	Paper wasp
Pol d 5	Antigen 5	
	Fruits	
Mal d 1	PR-10	Apple
Mal d 3	nsLTP	
Pers a	(Extract)	Avocado
Pers a 1	Class I chitinase	
Mus a 2		Banana
Mus a 5	β-1,3-glucanase	
Pru av 3	nsLTP	Cherry
Coc n 1	7/8S globulin	Coconut
Fic c	(Extract)	Fig
Vit v 1	nsLTP	Grape
Act d 1	Cysteine protease	Kiwi
Act d 2	TLP	
Act d 5	Kiwellin	
Act d 10	nsLTP	
Man i 1	Class IV chitinase	Mango
Cuc m 2	Profilin	Muskmelon
Car p	(Extract)	Papaya
Pru p 3	nsLTP	Peach
Pru p 7	Gibberellin-regulated protein	
Pyr c	(Extract)	Pear

Allergen/Extract	Allergen family	Allergen source
Fra a 3	nsLTP	Strawberry
	Vegetables	
Api g 1	PR-10	Celery
Api g 2	nsLTP	Celery
Api g 6	nsLTP	Celery
Api g 7	Plant defensin	Celery
All s	(Extracts)	Garlic
All c	(Extracts)	Onion
Sol t	(Extracts)	Potato
Sola l	(Extracts)	Tomato
Sola l 6	nsLTP	Tomato
	Grains	
Hor v	(Extracts)	Barley
Fag e	(Extracts)	Buckwheat
Fag e 2	2S albumin	Buckwheat
Zea m	(Extract)	Corn, cereal
Zea m 14	nsLTP	Corn, cereal
Sec c_flour	(Extracts)	Cultivated rye
Lup a	(Extracts)	Lupine seed
Pan m	(Extracts)	Millet
Ave s	(Extracts)	Oat
Che q	(Extracts)	Quinoa
Tri s	(Extracts)	Spelt
Tri a 14	nsLTP	Wheat
Tri a 19	Ω-5-gliadin	Wheat
Tri a 36	LMW glutenin	Wheat
Tri a 37	α-purothionin	Wheat
Tri a aA_TI	α-amylase trypsin-inhibitor	Wheat
	Legumes	
Cic a	(Extract)	Chickpea
Len c 1	7/8S globulin	Lentil
Len c 3	nsLTP	Lentil
Pis s 1	7/8S globulin	Pea
Pis s 2	7/8S globulin	Pea
Pis s 3	nsLTP	Pea
Ara h 1	7/8S globulin	Peanut
Ara h 2	2S albumin	Peanut
Ara h 3	11S globulin	Peanut
Ara h 6	2S albumin	Peanut
Ara h 8	PR-10	Peanut
Ara h 9	nsLTP	Peanut
Ara h 15	Oleosin	Peanut
Ara h 18	Cyclophilin	Peanut
Gly m 4	PR 10	Soy
Gly m 5	7/8S globulin	Soy
Gly m 6	11S globulin	Soy

<table>
<tr><th>Allergen/Extract</th><th>Allergen family</th><th>Allergen source</th></tr>
<tr><td>Gly m 8</td><td>2S albumin</td><td>Soy</td></tr>
<tr><td colspan="3">Nuts and seeds</td></tr>
<tr><td>Pru du</td><td>(Extract)</td><td rowspan="2">Almond</td></tr>
<tr><td>Pru du 6</td><td>11S globulin</td></tr>
<tr><td>Ber e</td><td>(Extract)</td><td rowspan="2">Brazil nut</td></tr>
<tr><td>Ber e 1</td><td>2S albumin</td></tr>
<tr><td>Ana o 1</td><td>7/8S globulin</td><td rowspan="3">Cashew</td></tr>
<tr><td>Ana o 2</td><td>11S globulin</td></tr>
<tr><td>Ana o 3</td><td>2S albumin</td></tr>
<tr><td>Cor a 1.0401</td><td>PR-10</td><td rowspan="5">Hazelnut</td></tr>
<tr><td>Cor a 8</td><td>nsLTP</td></tr>
<tr><td>Cor a 9</td><td>11S globulin</td></tr>
<tr><td>Cor a 11</td><td>7/8S globulin</td></tr>
<tr><td>Cor a 14</td><td>2S albumin</td></tr>
<tr><td>Mac i</td><td>(Extract)</td><td rowspan="2">Macadamia</td></tr>
<tr><td>Mac i 1.0101 (28-76)</td><td>α-hairpinin</td></tr>
<tr><td>Car i</td><td>(Extract)</td><td rowspan="4">Pecan</td></tr>
<tr><td>Car i 1</td><td>2S albumin</td></tr>
<tr><td>Car i 2 (256-386)</td><td>7/8S globulin</td></tr>
<tr><td>Car i 4</td><td>11S globulin</td></tr>
<tr><td>Pin p</td><td>(Extract)</td><td rowspan="2">Pine nut</td></tr>
<tr><td>Pin p 1</td><td rowspan="2">2S albumin</td></tr>
<tr><td>Pis v 1</td><td rowspan="3">Pistachio</td></tr>
<tr><td>Pis v 2</td><td>11S globulin</td></tr>
<tr><td>Pis v 3</td><td>7/8S globulin</td></tr>
<tr><td>Pap s</td><td>(Extract)</td><td rowspan="2">Poppy seed</td></tr>
<tr><td>Pap s 1.0101 (27-846)</td><td>α-hairpinin</td></tr>
<tr><td>Cuc p</td><td rowspan="2">(Extracts)</td><td>Pumpkin seed</td></tr>
<tr><td>Ses i</td><td rowspan="2">Sesame</td></tr>
<tr><td>Ses i 1</td><td>2S albumin</td></tr>
<tr><td>Hel a</td><td>(Extract)</td><td rowspan="2">Sunflower seed</td></tr>
<tr><td>Hel a 3</td><td>nsLTP</td></tr>
<tr><td>Jug r 1</td><td>2S albumin</td><td rowspan="5">Walnut</td></tr>
<tr><td>Jug r 2</td><td>7/8S globulin</td></tr>
<tr><td>Jug r 3</td><td>nsLTP</td></tr>
<tr><td>Jug r 4</td><td>11S globulin</td></tr>
<tr><td>Jug r 6</td><td>7/8S globulin</td></tr>
<tr><td colspan="3">Spices</td></tr>
<tr><td>Sin a</td><td>(Extract)</td><td rowspan="2">Mustard</td></tr>
<tr><td>Sin a 1</td><td>2S albumin</td></tr>
<tr><td colspan="3">Egg</td></tr>
<tr><td>Gal d_white</td><td>(Extract)</td><td rowspan="5">Chicken egg white</td></tr>
<tr><td>Gal d 1</td><td>Ovomucoid</td></tr>
<tr><td>Gal d 2</td><td>Ovalbumin</td></tr>
<tr><td>Gal d 3</td><td>Ovotransferrin</td></tr>
<tr><td>Gal d 4</td><td>Lysozyme C</td></tr>
</table>

Allergen/Extract	Allergen family	Allergen source
Gal d_yolk	(Extract)	Chicken egg yolk
Gal d 5	Serum albumin	
Milk		
Cam d	(Extracts)	Camel's milk
Bos d_milk		Cow's milk
Bos d 4	α-lactalbumin	
Bos d 5	β-lactoglobulin	
Bos d 8	Casein	
Bos d 9	α-S1 casein	
Bos d 10	α-S2 casein	
Bos d 11	β-casein	
Bos d 12	κ-casein	
Cap h_milk	(Extracts)	Goat's milk
Equ c_milk		Mare's milk
Ovi a_milk		Sheep's milk
Seafood		
Ani s 1	Kunitz serine protease inhibitor	*Anisakis simplex*
Ani s 3	Tropomyosin	
Gad m 1	β-parvalbumin	Atlantic cod
Clu h	(Extract)	Atlantic herring
Clu h 1	β-parvalbumin	
Sco s	(Extract)	Atlantic mackerel
Sco s 1	β-parvalbumin	
Pen m 1	Tropomyosin	Black tiger shrimp
Pen m 2	Arginine kinase	
Pen m 3	Myosin light chain	
Pen m 4	Sarcoplasmic calcium-binding protein	
Cra c 6	Troponin C	Brown shrimp
Cyp c 1	β-parvalbumin	Carp
Cyp c 2	β-enolase	
Chi spp.	(Extract)	Crab
Mac r 1	Tropomyosin	Giant freshwater prawn
Mac r 2	Arginine kinase	
Hom g	(Extracts)	Lobster
Pan b		Northern prawn
Sal s		Salmon
Sal s 1	β-parvalbumin	
Sal s 6	Collagen	
Lit spp.	(Extracts)	Shrimp mix
Lol spp.		Squid
Xip g 1	β-parvalbumin	Swordfish
Raj c	(Extract)	Thornback ray
Raj c Parvalbumin	α-parvalbumin	
Thu a 1	β-parvalbumin	Tuna
Rud spp.	(Extract)	Venus clam

Allergen/Extract	Allergen family	Allergen source
Lit v 7	Haemocyanin	Whiteleg shrimp
	Meat	
Bos d_meat	(Extract)	Beef
Bos d 6	Serum albumin	
Gal d_meat	(Extract)	Chicken
Gal d 7	Myosin light chain	
Equ c_meat	(Extracts)	Horse
Ach d		House cricket
Ten m		Mealworm
Loc m		Migratory locust
Sus d 1	Serum albumin	Pork
Ory a_meat	(Extract)	Rabbit
Galactose-α-1,3-galactose	α-gal	Red meat
Ovi a_meat	(Extract)	Lamb
Mel g		Turkey
	Others	
Hom s LF	CCD-marker	Hom s lactoferrin
Hev b 1	Rubber elongation factor	Latex
Hev b 3	Small rubber particle protein	
Hev b 5	Unknown	
Hev b 6.02	Pro-hevein	
Hev b 11	Class I chitinase	
Arg r 1	Lipocalin	Pigeon tick
Fic b	(Extract)	Weeping fig

Figure S1

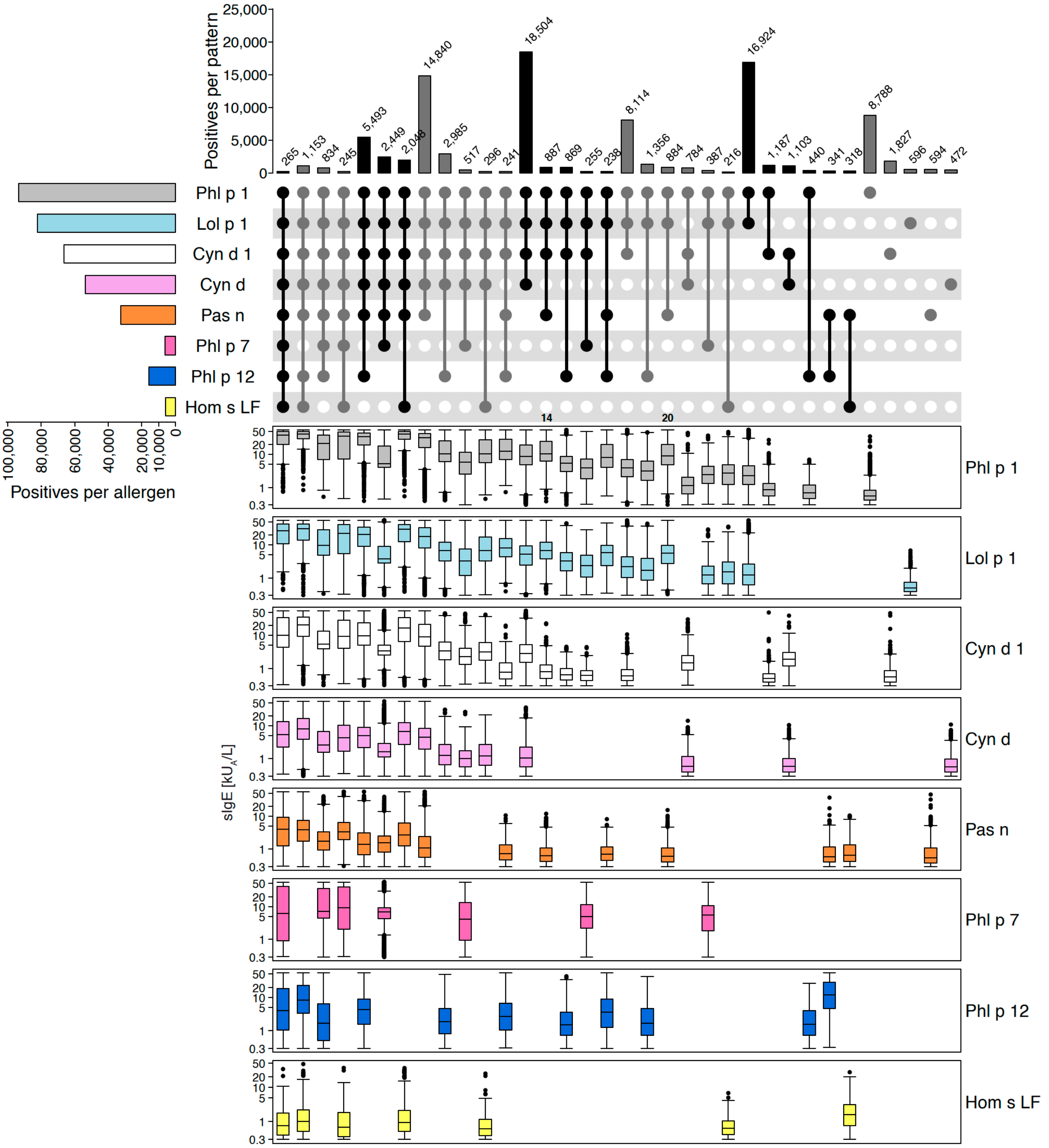

Figure S1. Combined Upset-plot and box-plot for group 1 grass pollen allergens (Phl p 1, Lol p 1, Cyn d 1), extracts from Bermuda grass (Cyn d) and Bahia grass (Pas n), the pan-allergen families of polcalcins (Phl p 7) and profilins (Phl p 12), and for CCDs (Hom s LF). Upset-plot (top), showing different reactivity patterns to grass pollen allergens and extracts, and to surrogate markers of the pan-allergen families of polcalcins (Phl p 7) and profilins (Phl p 12), as well as to a CCD-marker (Hom s LF). Numbers of cases for each individual allergen are indicated by bars on the left ("Positives per allergen"), and for each combination of IgE-reactivity by bars on top of the plot ("Positives per pattern"). The combination matrix illustrates patterns of reactivity: a single dark (i.e., black or dark grey) dot represents samples monoreactive to the respective allergen on the left, two dark dots that are connected by a vertical line show samples with IgE-reactivity to the two corresponding allergens on the left side, etc. To facilitate readability, every second line is shown in light grey; white dots display absence of reactivity. Groups of reactivities

to the same number of allergens are sorted from left (highest number of co-reactivities) to right (monoreactivities), each group either displayed by black or grey bars and dots, to facilitate identification of groups. At the bottom of the combination matrix, select columns mentioned in the text are indicated by numbers. Box-plots underneath each individual reactivity pattern show IgE-levels (kU_A/L) to each of the abovementioned allergen-preparations (y-axes in log_{10} scale).

Figure S2

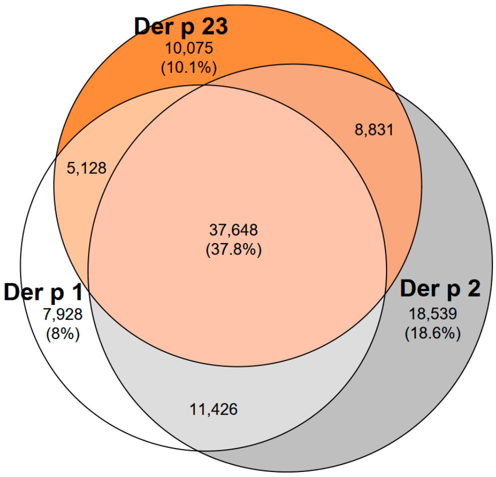

Figure S2. Co-reactivity of Der p 1, Der p 2 and Der p 23. Euler-plot of all samples positive to at least one of the HDM-specific marker allergens Der p 1, Der p 2 or Der p 23 (n=99,575) on ALEX[2]. Absolute numbers of cases are indicated for each respective segment. For selected segments, the percentage share of all cases is shown.
